# Supplementary material for: A Comprehensive Assessment of Ultraviolet-Radiation-Induced Mutations in Flammulina filiformis Using Whole-Genome Resequencing
Source: J Fungi (Basel). 2024 Mar 20;10(3):228. doi: 10.3390/jof10030228 (PMC10971301; doi:10.3390/jof10030228)
Supplement: Supplementary file 1 [file jof-10-00228-s001.zip › Supplementary Material S8/KEGG annotation/out/64381550635650.os/KO/out_map/map04530.html]

KEGG PATHWAY: Tight junction - Reference pathway


|  |  |
| --- | --- |
| **Tight junction - Reference pathway** |  |

[
Pathway menu
| Organism menu
| Pathway entry
| Show description
| User data mapping
]

Epithelial tight junctions (TJs) are composed of at least three types of transmembrane protein -occludin, claudin and junctional adhesion molecules (JAMs)- and a cytoplasmic 'plaque' consisting of many different proteins that form large complexes. The transmembrane proteins mediate cell adhesion and are thought to constitute the intramembrane and paracellular diffusion barriers. The cytoplasmic 'plaque' contains three major multi-protein complexes consisting largely of scaffolding proteins, the ZO protein complex, the CRB3-Pals1-PATJ complex and the PAR-3-aPKC-PAR-6 complex. The ZO protein complex appears to organize the transmembrane proteins and couple them to other cytoplasmic proteins and to actin microfilaments. Two evolutionarily conserved protein complexes, the CRB3 and PAR complexes are involved in the establishment and maintenance of epithelial cell polarity. Besides these three protein complexes which seem to be constitutively associated at TJs, a number of proteins with different functions has been identified at TJs. These include additional scaffolding proteins like MUPP1 and MAGI-1, adaptor proteins, transcription regulators and RNA processing factors, regulatory proteins like small GTPases and G-proteins, kinases and phosphatases, and heat shock proteins. These are proposed to be involved in junction assembly, barrier regulation, gene transcription, and perhaps other, presently undefined pathways.

|  |  |
| --- | --- |
| Reference pathway | 100% |
